# Correction of vitamin D deficiency in critically ill patients - VITdAL@ICU study protocol of a double-blind, placebo-controlled randomized clinical trial

**DOI:** 10.1186/1472-6823-12-27

**Published:** 2012-11-07

**Authors:** Karin Amrein, Christian Schnedl, Andrea Berghold, Thomas R Pieber, Harald Dobnig

**Affiliations:** 1 Division of Endocrinology and Metabolism, Department of Internal Medicine, Medical University of Graz, Auenbruggerplatz 15, Graz A-8036, Austria; 2 Institute for Medical Informatics, Statistics and Documentation, Medical University Graz, Graz, Austria

**Keywords:** Critical Illness, Vitamin D deficiency, Cholecalciferol, Vitamin D, Critical care, Intensive care, Vitamin D3

## Abstract

**Background:**

Vitamin D deficiency is associated with multiple adverse health outcomes including increased morbidity and mortality in the general population and in critically ill patients. However, no randomized controlled trial has evaluated so far whether treatment with sufficiently large doses of vitamin D can improve clinical outcome of patients in an intensive care setting.

**Methods/design:**

The VITdAL@ICU trial is an investigator-initiated, non-commercial, double-blind, placebo-controlled randomized clinical trial. This study compares high-dose oral cholecalciferol (vitamin D3) versus placebo treatment in a mixed population of 480 critically ill patients with low 25-hydroxyvitamin-D levels at study enrollment (≤ 20ng/ml). Following an initial loading dose of 540,000 IU of vitamin D3, patients receive 90,000 IU of vitamin D3 on a monthly basis for 5 months. The study is designed to compare clinical outcome in the two study arms with the primary endpoint being length of hospital stay. Secondary endpoints include among others length of ICU stay, the percentage of patients with 25(OH)D levels > 30 ng/ml at day 7, ICU and hospital mortality and duration of mechanical ventilation. We describe here the VITdAL@ICU study protocol for the primary report.

**Discussion:**

This trial is designed to evaluate whether high-dose vitamin D3 is able to improve morbidity and mortality in a mixed population of adult critically ill patients and correct vitamin D deficiency safely.

**Trial registration:**

ClinicalTrials: NCT01130181

## Background

Vitamin D is a key regulator in calcium and phosphorus metabolism and likely confers physiologically relevant pleiotropic functions that include cardioprotective and immunomodulatory effects as well as improvement of antimicrobial action [1-6]. Profound vitamin D deficiency in humans causes rickets in children or osteomalacia in adults together with vitamin D myopathy, whereas low vitamin D levels may be associated with an increased prevalence of a multitude of other diseases [1,6] including an increase in mortality [7,8].

Moreover, a systematic review concluded that intervention with cholecalciferol significantly reduced mortality in predominantly elderly, institutionalized women [9]. To date, only few large interventional randomized trials have investigated the effect of vitamin D on clinical outcomes. The rationale for pleiotropic effects of this substance is based on the fact that the vitamin D receptor (VDR) is almost ubiquitously expressed, and the vast majority of cells respond to 1,25(OH)2D exposure. Hundreds of genes with vitamin D receptor response elements directly or indirectly influence cell cycling, cell proliferation, differentiation and apoptosis [6,10].

### Vitamin D in ICU patients

Endogenous synthesis in the presence of UV-B radiation is the main source of vitamin D. Therefore, immobilized and elderly individuals are prone to develop vitamin D deficiency. Low vitamin D levels are highly prevalent in critically ill adults and children [11-18]. Unfortunately, no uniform definition of vitamin D deficiency exists and tissue levels of the major active metabolite 1,25(OH)2D are currently not measurable. Today most guidelines agree, however, that a “low” vitamin D status is present when serum 25(OH)D levels are below 20 ng/ml [1,19]. Special concerns in the ICU setting relate to the issue as to whether the definition of vitamin D deficiency should be the same as in the general population or whether special metabolites of vitamin D should be considered in this definition as well [20]. Insufficient vitamin D levels could affect this population in various ways [21]. Over the last years, several groups have reported on an inverse association between vitamin D levels in critically ill patients and severity of disease including length of ICU stay and mortality [13,14,16,22,23]. Moreover, vitamin D levels decline further during ICU stay [24] which is explained by insufficient replacement of vitamin D via enteral or parenteral nutrition in the absence of UV-B exposure. To date, only few and small intervention studies (n=22-33 patients) that were not designed to demonstrate effects on clinical outcomes have been published [12,25-27].

### Vitamin D and proposed clinical effects pertinent to the critically ill patient

#### Calcium homeostasis and bone turnover

In critically ill patients, hypocalcemia is frequently present and has been related to increased morbidity and mortality [28-30]. Reasons for hypocalcemia are not well understood, but besides elevated levels of IL-6, vitamin D deficiency may play a decisive role [31-34]. Without correction of vitamin D deficiency, calcium levels may be impossible to normalize [29]. To maintain serum calcium levels, parathyroid hormone levels rise and induce accelerated bone resorption which is aggravated by prolonged immobilization ultimately resulting in significant bone loss. Interestingly, even relatively low doses of intravenous vitamin D (500 IU daily) might be able to attenuate this detrimental physiological response [12].

#### Cardiovascular system

The heart is an important target organ for vitamin D, both on a genomic and nongenomic level [35]. Cardiomyocytes express the VDR. Severe hypocalcemia may cause life-threatening, reversible heart failure [36]. Vitamin D seems to inhibit activation of the renin-angiotensin system as well as the expression of genes involved in the development of myocardial hypertrophy [37]. There is accumulating evidence that vitamin D deficiency may be an important factor in the development of congestive heart failure and sudden cardiac death [38]. In chronic hemodialysis patients, calcitriol supplementation is associated with reduction of cardiac hypertrophy and a reduction of QT dispersion [39], the latter being considered a major risk factor for arrhythmia and sudden cardiac death.

Our group found a negative correlation of 25(OH)D with NT-pro-BNP levels, NYHA functional classes and impaired left ventricular function [38]. Hazard ratios for death attributable to heart failure and sudden cardiac death were 2.8 and 5.0, respectively, when patients with severe vitamin D deficiency (25(OH)D <10 ng/ml) were compared with those considered having optimal serum levels (25(OH)D >30 ng/ml) [38]. The anti-inflammatory properties of vitamin D may play an additional role in congestive heart failure [40]. In animal models, vitamin D deficiency is associated with myocardial hypertrophy and fibrosis leading to aberrant cardiac contractility and relaxation [41,42]. Moreover, calcitriol treatment was able to prevent cardiac hypertrophy under in-vitro conditions or in VDR knock-out animals [41,42].

#### Immune system/sepsis/inflammation

Vitamin D acts on both the adaptive and innate immunity systems. Most cells of the immune system express the VDR [6]. It enables macrophages to respond to and kill bacterial and viral organisms. In line with this is the observation of impaired macrophage function in VDR knock-out mice [6]. Vitamin D also exerts an influence on epidermal keratinocytes to fulfill their barrier function [43]. Monocytes cultured in severely vitamin D-deficient plasma express less cathelicidines and improve their function with 25(OH)D as well as 1,25(OH)2D [44]. Cathelicidines are important antimicrobial peptides that are of great interest in critically ill patients because they constitute part of the initial defense line against pathogens. They are expressed in epithelia of airways, the bladder and gastrointestinal tract, which are all considered important entry sites of nosocomial infections. Treatment of normal human bronchial epithelial cells with 1,25(OH)2D results in a 10-fold up-regulation of cathelicidin mRNA levels after 12 hours [45]. In septic ICU patients, a positive correlation between circulating 25(OH)D and LL-37 levels, the active fragment of cathelicidin, has been reported [46]. In a large retrospective study, the risk for blood culture positivity was significantly higher in vitamin D deficient critically ill patients [13]. Of interest in this context is also the finding that sepsis incidence and case-fatality rates are significantly higher in winter despite similarities in disease severity during summer time [47].

#### Muscle

Severe vitamin D deficiency is associated with myopathy, decreased muscle strength and an increased risk for falls [1]. In intensive care, this may translate to the necessity of prolonged mechanical ventilation and difficulties during remobilization and weaning from ventilatory support in patients with low vitamin D status. In randomized controlled trials, vitamin D supplementation improved muscle strength in elderly patients [48] and decreased the frequency of falls [48-50].

### Guidelines and practices

For the general population, the Institute of Medicine (IOM) recommends a daily allowance for adults of 600 to 800 IU [51], while the Endocrine Society Guideline recommends 1,500–2,000 IU of vitamin D3 per day for the at-risk population [52]. Upper daily limits for adults are 4,000 (IOM)–10,000 (Endocrine Society) IU per day. Guidelines for parenteral nutrition recommend 200 IU per day and typical enteral nutrition formulas contain 200–300 IU of ergocalciferol or cholecalciferol per 1000 ml. Available over the counter multivitamin preparations commonly contain 200 IU of vitamin D2 or D3. Currently, there is no intravenous vitamin D-monopreparation available, although high-dose intramuscular preparations may be an option in selected patients.

### Rationale of the study and hypothesis

In summary, vitamin D plays a pivotal role in calcium homeostasis and is, based on current knowledge, associated with disease severity and mortality in critically ill patients. Moreover, vitamin D exhibits a number of pleiotropic effects that are of special interest to the ICU patient [20,53,54] However, so far it is unclear whether a low vitamin D status is causally linked to adverse outcomes or merely an epiphenomenon reflecting poor health status. In contrast to previous clinical trials that failed to show a benefit for correcting hormonal insufficiencies in critical illness (e.g. administration of growth hormone or steroids) [55,56], one has to bear in mind that vitamin D not only acts on endocrine, but also autocrine level which allows for tissue-specific control of 1,25(OH)2D regulation and action that may be completely independent of circulating calcitriol levels. As far as we know now vitamin D also has an excellent safety profile with a broad therapeutic window [19,57].

The hypothesis of this first large RCT is that supplementation with a sufficiently large dose of vitamin D leads to a fast correction of low vitamin D status and possibly to clinical benefits, particularly relating to immune, cardiac and muscle function. Because these potential benefits likely occur at various functional hierarchies, we chose “length of hospital stay” as a primary surrogate marker for a change in morbidity in this patient group.

### Results from the pilot study relevant for the design of this trial

To optimize study design and ascertain safety and efficacy of the chosen loading dose of 540,000 IU of cholecalciferol, we performed a pilot trial in 25 medical critically ill patients with vitamin D deficiency [25]. Adverse effects like hypercalcemia or hypercalciuria did not occur in this pilot study and vitamin D levels increased significantly in most patients within two days, although 2 out of 10 patients showed no or only a modest rise in 25(OH)D levels at day 7. We therefore chose to continue with the same loading dose, but added monthly maintenance doses of vitamin D to the study protocol.

### Justification of high-dose vitamin D3 intervention

The half-life of 25(OH)D following oral cholecalciferol supplementation allows the administration of large doses. In elderly patients with vitamin D deficiency and healthy individuals, large loading doses rapidly and safely normalized 25(OH)D levels [58-60]. However, an annual high-dose intervention was associated with a higher risk of falls and fractures in elderly patients [61]. Some contraindications such as sarcoidosis and other granulomatous diseases also need to be considered before administration of a high loading dose [62,63]. Following publication of the detrimental effects of a high-dose vitamin D intervention on the risk of falls and fractures [61], we decided to nevertheless proceed with the initial study protocol because in our population, a rapid restoration of vitamin D status may still have positive effects on patient immunity and even muscular function. We are aware that smaller doses of vitamin D3 are likely an effective treatment option for most patients with low vitamin D status within months but this time span may be too long during critical illness.

## Methods/design

### Study design

The VITdAL@ICU trial is an investigator-initiated, non-commercial, double-blind, placebo-controlled randomized clinical trial. This study compares high-dose oral cholecalciferol (vitamin D3) versus placebo treatment in a mixed population of 480 critically ill patients with low 25(OH)D levels at study enrollment (≤ 20ng/ml). Following an initial loading dose of 540,000 IU of vitamin D3, patients receive 90,000 IU of vitamin D3 on a monthly basis for 5 months resulting in a total treatment/observation period of 6 months. The VITdAL@ICU study is conducted at the Medical University of Graz, Austria, a tertiary care university hospital with a catchment area covering the Southeast of Austria (population approximately 1.5 million). It recruits patients from five ICUs: medical (15 beds), neurological (8 beds), cardiothoracic surgery (13 beds) and two mixed surgery units (12 and 10 beds, respectively).

### Eligibility criteria for inclusion and recruitment

Upon admission to one of the five participating ICUs, all adult patients undergo screening for study participation. Patients ≥18 years who are expected to stay at the ICU ≥48 hours and are vitamin D deficient (25(OH)D level ≤ 20 ng/ml) are screened for inclusion and exclusion criteria. The following patients are not considered eligible for inclusion: patients expected to die within the upcoming 24 hours, with severely impaired gastrointestinal motility (ileus, gastric volume > 400 ml), contraindication for study drug application (orally or via feeding tube), readmission after participation in the VITdAL@ICU pilot study, enrolment in another intervention trial or documented hypercalcemia (total calcium >2.65 or ionized serum calcium >1.35 mmol/l). Patients with a history of granulomatous disease (tuberculosis, sarcoidosis), recent kidney stones (≤ 1 year) as well as pregnant or lactating women are also excluded from this trial. An overview of the study procedures is given in Figure 1.

**Figure 1:**
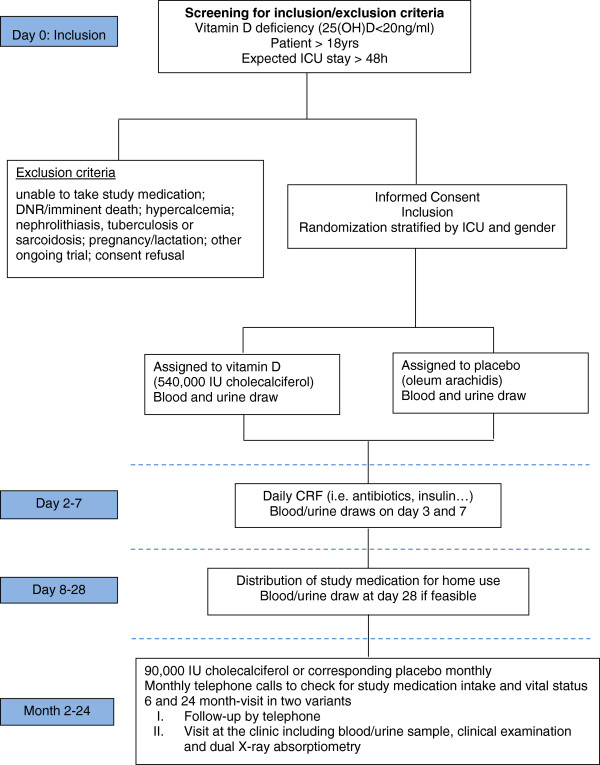
Trial procedures flow sheet.

### Ethical aspects and informed consent

The institutional ethical committee of the Medical University of Graz, Austria (reference number 21–214 ex 09/10, EudraCT-Nr.: 2010-018798-39), and the Austrian Agency for Health and Food Safety (AGES) approved the study. Informed consent is obtained prior to inclusion into the study according to the Austrian law. The patient can withdraw from the study at any time, without giving any reason and without impact on treatment. A register is kept of all patients evaluated for inclusion and of patients who choose to withdraw from the study.

### Randomization

Patients at each of the five ICUs are randomly assigned to one of the two treatment study groups, “Vitamin D” or “Placebo”, in a 1:1 ratio, using the web-based randomization tool “Randomizer for Clinical Trials” ( http://www.randomizer.at). Block randomization is used with patients stratified according to ICU and gender.

### Study drug preparation, labeling and intake

The cholecalciferol preparation is provided by Fresenius Kabi, Austria. As placebo, oleum arachidis is used. Identical bottles of study medication containing 180,000 IU of cholecalciferol in 15 ml of oleum arachidis or corresponding placebo are prepared and labeled at the institutional pharmacy. Study medication is stored at room temperature. For usage of the study medication outside of the ICU, the patient, next of kin or continuing caregiver/s are instructed by the study team and receive oral and written information on how and when to take the remaining study medication. Furthermore, they are asked to refrain from additional use of vitamin D over the following 6 months. Additionally, the patient’s family practitioner and, if applicable, treating general ward or rehabilitation facility are personally informed or contacted by telephone and instructed about the study.

### Interventions

“Vitamin D”: Patients randomized to the “Vitamin D” group receive a loading dose of 540,000 IU of vitamin D3 orally or via feeding tube. This dose equals three bottles of a commercially available vitamin D preparation in Austria: Oleovit D3® solution; concentration of cholecalciferol is 400 IU vitamin D3/drop of oleum arachidis. Total carrier volume is 45 ml. From month 1 to 5, patients receive monthly maintenance doses of 90,000 IU cholecalciferol.

“Placebo”: Patients randomized to the “Placebo” group receive oleum arachidis in the same volume and time schedule as “Vitamin D” patients.

### Routine low-dose vitamin D supplementation in both study arms during ICU stay

Patients in both study arms are allowed to receive regular low dose (approximately 200 IU per day) vitamin D supplements via enteral and/or parenteral nutrition as deemed appropriate by the treating physicians during stay at the ICU, but are not allowed to receive any vitamin D preparations during their stay at the general ward or at home.

### Handling of readmissions to ICU

Patients who are readmitted to intensive care after participation in the VITdAL@ICU trial are not eligible for reinclusion.

### Blinding of treatment allocation

All persons involved in patient care of the RCT are blinded to treatment allocation and all study-related activities are carried out in a double-blind fashion.

### Primary outcome measures

The primary efficacy endpoint for this RCT is the length of stay (LOS) in the hospital starting from application of the study medication. The end is defined as the time of hospital discharge or death of the patient. Further admissions to rehabilitation facilities are not taken into account. Before unblinding all relevant facilities are independently classified by two study team members.

### Secondary outcome measures

Secondary endpoints for this RCT include:

• length of ICU stay starting from application of study medication

• ICU and hospital mortality, 28-day mortality, 6-month mortality

• percentage of patients with 25(OH)D levels ≥ 30 ng/ml at day 7

• duration of mechanical ventilation including CPAP/mask ventilation starting from application of study medication

• need for a tracheostomy during ICU stay

• surrogate markers for cardiac function: need for vasopressor therapy during ICU stay, and duration of such need, NT-proBNP levels

• analysis of biochemical and endocrine variables relevant to calcium- and phosphorus metabolism; 1,25(OH)D and PTH at days 0,3,7, 28 and month 6

• C-Reactive Protein and procalcitonin levels

• clinical parameters at 6 months (falls, Timed Up & Go – test, hand grip strength, lumbar and hip BMD, Quality of life and performance status, number of hospitalizations, number of respiratory tract infections)

• serum cathelicidin levels at day 0 and 7 are planned to be analyzed from frozen samples and will be reported separately

### Data collected at study entry

At baseline, data on demographic and clinical characteristics of the patients are obtained. Simplified Acute Physiology Score (SAPS II), presence of comorbidities, medical history, admission diagnosis by category and relevant medication are registered.

The following categories for diagnosis at admission and concomitant diagnoses are predefined:

• Surgical ICU admissions:

• trauma, cardiac surgery, vascular surgery, thoracic surgery, brain surgery, transplantation, other surgical reasons

• Medical or neurological ICU admissions:

• cardiovascular, respiratory, hematological/oncological, sepsis/infectious, gastrointestinal/hepatic, renal, metabolic, neurological, other non-surgical reasons

In addition, we record the need for and the number of days of hemodynamic support and mechanical ventilation including noninvasive ventilation and tracheostomy as well as the quantity and formula of enteral/parenteral nutrition given. Information on insulin requirements and use of antibiotics are registered on day 0, 2 and 6.

### Blood and urinary samples

Venous blood samples are taken upon ICU admission and in the morning on days 0,3,7,28 if feasible. Analyses include routine serum chemistry, hematology, markers of inflammation and parameters of calcium and vitamin D metabolism. Additional measurements of markers of metabolism and inflammation as well as other endocrine parameters are planned on stored samples that are frozen and stored at −70°C.

### 25(OH)D assay

25(OH)D serum levels are measured by ELISA (Immunodiagnostic Systems, Boldon, UK).

### Reminder calls and follow-up procedures

All patients who leave the hospital are contacted by telephone on a monthly basis to check for compliance with the monthly intake of the study drug (5 times) and vital status.The long-term follow-up appointment at 6 and 24 months is performed in two ways, dependent on the patient’s mobility and preference:

• Variant A (estimated 80%): Follow-up visit by telephone

• Variant B (estimated 20%): Visit at the clinic, including additional:

• Blood and urinary testing (similar to that on days 0,3,7,28)

• Timed Up & Go – Test (in seconds)

• Hand grip strength (in mmHg)

• Dual X-ray absorptiometry (DXA) with bone mineral density (at the hip and the lumbar spine) and body composition

The following data set is collected in all patients:

• Compliance with intake of study drug (number of maintenance doses)

• Additional vitamin D intake

• Falls and fractures since study inclusion (number)

• Respiratory tract infections (number and use of antibiotics)

• Hospitalizations (number and cause)

• Performance status (Eastern Cooperative Oncology Group (ECOG) Score)

• Quality of life

### Data management

Data is manually transferred from source files into paper-based primary CRFs identified by ascending patient numbers specific to respective ICUs. Data are then checked for accuracy and transferred to an electronic CRF by the clinical research assistance team. Extensive verification checks are performed by a study monitor. Vital status at 6 months is recorded for all patients.

### Safety assessment

Blinded safety assessment for 28-day mortality is planned after 100 and 240 patients. In case there is a safety concern, a standby data safety monitoring board meeting is planned.

### Sample size calculation

The sample size was calculated to detect a difference in mean length of hospital stay of 2 days with 80% power and a significance level of 5%. Using the Mann–Whitney U-Test for sample size calculation, a group size of 234 is needed to show a reduction of 2 days (equivalent to 14%) of the primary study outcome based on a mean hospital stay of 14 days and standard deviation of 7 days for the control group. To consider drop-outs of the study, a sample of 480 patients (240 per arm) was considered appropriate.

### Statistical analyses

The primary analysis for comparing length of hospital stay between the two groups will be made using the Mann–Whitney U-test. For secondary endpoints, differences between the two groups will be evaluated with the t test or Mann–Whitney U-test for continuous variables as appropriate and for categorical variables with the chi-square or Fisher Exact test. Survival endpoints will be displayed in Kaplan-Meier plots and compared by the log rank test. Furthermore, linear and logistic regression models will be applied to adjust for age, disease severity and 25(OH)D.

A CONSORT diagram will be reported (Figure 2). Analyses will be performed both on intention to treat and per protocol population with the intention-to-treat population as the primary. To assess compliance with the study protocol, the number of study drug maintenance doses will be reported.

**Figure 2:**
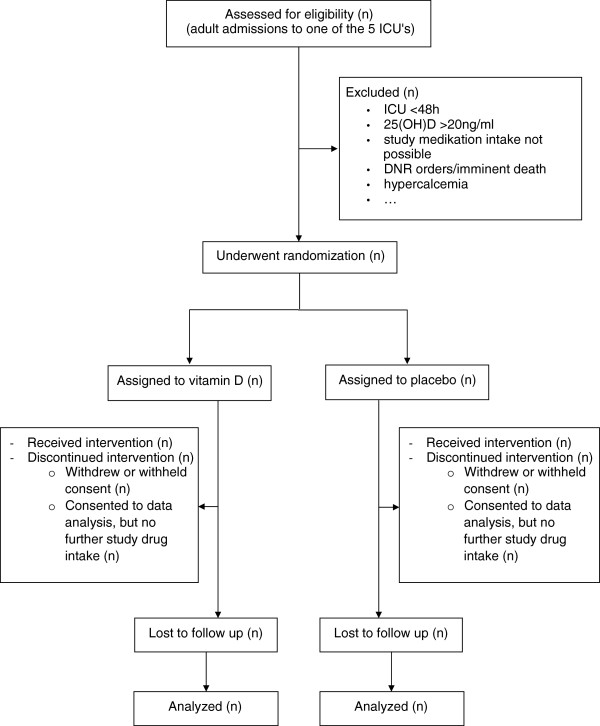
**CONSORT flow diagram.** All adult patients admitted to five intensive care units (ICU; medical, neurological, cardiac surgery and two mixed surgery units) from May 4, 2010 onward were eligible for inclusion.

### Safety endpoints

Safety endpoints comprise vital status (ICU and hospital mortality, 28-day mortality, 6-month mortality) as well as serum 25(OH)D and calcium levels. 25(OH)D and serum calcium levels are measured at day 0, 3, 7 and, if available, at day 28 and month 6 in order to identify vitamin D intoxication and/or hypercalcemia. Following the report by Sanders et al. of a higher risk of falls and fractures in elderly women on an annual high-dose vitamin D regime in May 2010 [61], we decided to add the number of self-reported falls as additional safety endpoint.

## Discussion

The study has been initiated as planned on May 04 2010. Blinded safety analyses for 28-day mortality after 100 and 240 patients did not reveal a difference between the two study groups.

This study is the first large RCT evaluating the role of vitamin D on clinical outcomes in critically ill patients. Therefore, a significant difference in the safety and/or efficacy endpoints will provide important evidence for or against the use of high-dose vitamin D intervention in an intensive care setting and, in the event of a favorable outcome in the intervention group, aid in the design of future larger RCTs adequately powered to detect a possible difference in mortality in ICU patients and possibly hospitalized patients in general.

## Abbreviations

25(OH)D: 25-hydroxyvitamin D; 1,25(OH)2D: 1,25-dihydroxyvitamin D; BMI: Body Mass Index; CRF: Case Report Form; LOS: Length of stay; ICU: Intensive Care Unit IOM: Institute of Medicine; IU: International units; NYHA: New York Heart Association Functional Class RCT: Randomized Clinical Trial; SD: Standard Deviation.

## Competing interests

The funding for this trial was provided by the Austrian National Bank (Jubiläumsfonds, Project Nr. 14143), a grant from the European Society for Clinical Nutrition and Metabolism (ESPEN) and an institutional, unconditional and non-restrictive research grant from Fresenius Kabi, Austria. The sponsors had no influence on study design, patient recruitment or data generation and will not have any impact on the analysis of the results or writing of the manuscript. HD received lecture fees from Fresenius Kabi, Austria and KA received lecture fees from BBraun, Germany and NovoNordisk, Denmark. All other authors have no financial or non-financial competing interests relevant to this study to declare.

## Authors’ contributions

KA and HD designed the VITdAL@ICU study protocol. KA, CS and HD drafted the manuscript and AB is responsible for sample size calculation and statistical analyses. For the primary report of the clinical results, the authorship will be determined by intellectual and operational contribution. All authors read and approved the final manuscript.

## Pre-publication history

The pre-publication history for this paper can be accessed here:

http://www.biomedcentral.com/1472-6823/12/27/prepub
